# The Initiation Factor TFE and the Elongation Factor Spt4/5 Compete for the RNAP Clamp during Transcription Initiation and Elongation

**DOI:** 10.1016/j.molcel.2011.05.030

**Published:** 2011-07-22

**Authors:** Dina Grohmann, Julia Nagy, Anirban Chakraborty, Daniel Klose, Daniel Fielden, Richard H. Ebright, Jens Michaelis, Finn Werner

**Affiliations:** 1 University College London, Institute for Structural and Molecular Biology, Division of Biosciences, Darwin Building, Gower Street, London WC1E 6BT, UK; 2 Department of Chemistry and Center for Integrated Protein Science München, Ludwig-Maximilians-Universität München, Butenandtstrasse11, 81377 München, Germany; 3 Howard Hughes Medical Institute, Rutgers University, Piscataway, NJ 08902, USA; 4 Waksman Institute, Rutgers University, Piscataway, NJ 08902, USA; 5 Department of Chemistry and Chemical Biology, Rutgers University, Piscataway, NJ 08902, USA

## Abstract

TFIIE and the archaeal homolog TFE enhance DNA strand separation of eukaryotic RNAPII and the archaeal RNAP during transcription initiation by an unknown mechanism. We have developed a fluorescently labeled recombinant *M. jannaschii* RNAP system to probe the archaeal transcription initiation complex, consisting of promoter DNA, TBP, TFB, TFE, and RNAP. We have localized the position of the TFE winged helix (WH) and Zinc ribbon (ZR) domains on the RNAP using single-molecule FRET. The interaction sites of the TFE WH domain and the transcription elongation factor Spt4/5 overlap, and both factors compete for RNAP binding. Binding of Spt4/5 to RNAP represses promoter-directed transcription in the absence of TFE, which alleviates this effect by displacing Spt4/5 from RNAP. During elongation, Spt4/5 can displace TFE from the RNAP elongation complex and stimulate processivity. Our results identify the RNAP “clamp” region as a regulatory hot spot for both transcription initiation and transcription elongation.

## Graphical abstract

**Graphical Abstract:**

**Figure:**
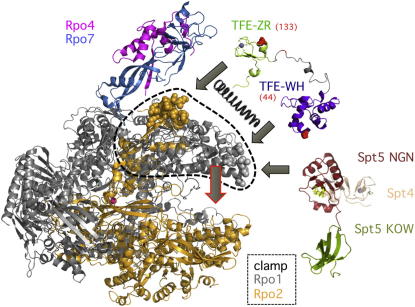

**Highlights:**

► The RNAP clamp coiled coil and RNAP stalk are required for TFE binding and activity ► The elongation factor Spt4/5 can inhibit PIC formation and transcription initiation ► TFE efficiently prevents inhibition of transcription initiation by Spt4/5 ► Spt4/5 displaces TFE from the TEC

## Introduction

RNA polymerases (RNAPs) are responsible for DNA-dependent transcription in all living organisms (Jun et al., 2011; Werner and Grohmann, 2011). In contrast to eukaryotes, who employ between three (animal) and five (plant) distinct nuclear RNAPs to transcribe distinct and nonoverlapping subsets of genes, archaea only have one RNAP. However, the subunit composition of the archaeal RNAP, its structure, and its requirements for general transcription factors bear close resemblance to those of eukaryotic RNAPII (Werner and Grohmann, 2011). The archaeal RNAP system offers substantial experimental advantages over the eukaryotic counterparts. Thus, it is possible to reconstitute an archaeal RNAP from its 12 individual recombinant subunits in vitro under defined conditions, a feat that has not been achieved in any eukaryotic system to date (Naji et al., 2007; Werner and Weinzierl, 2002). The ability to reconstitute archaeal RNAP in vitro has enabled us to site-specifically introduce molecular probes into separate RNAP subunits with the aim of characterizing dynamic properties of transcription complexes (Grohmann et al., 2010).

In eukaryotes and archaea, TBP and TFIIB (TFB in archaea) are necessary and sufficient to direct transcription initiation from strong promoters in vitro (Parvin and Sharp, 1993; Qureshi et al., 1997; Werner and Weinzierl, 2002). A third evolutionary conserved factor, TFIIE (TFE in archaea), is not strictly required, but stimulates initiation by enhancing DNA strand separation (Forget et al., 2004; Naji et al., 2007) and in eukaryotes by aiding the recruitment of the RNAPII-specific transcription factor TFIIH (Holstege et al., 1995; Holstege et al., 1996). TFIIE (TFE) homologs can be found in several different RNAP systems. For example, eukaryotic RNAPIII includes two subunits, C82 and C34, that are homologous to TFIIEα and β, respectively (Geiger et al., 2010; Carter and Drouin, 2010). Archaeal TFE consists of two principal domains, a winged helix (WH) and a Zinc ribbon (ZR) domain, which together are homologous to the N-terminal part of the eukaryotic TFIIEα subunit (Bell et al., 2001). In yeast the corresponding region of the TFIIEα subunit is sufficient for TFIIE activity (Kuldell and Buratowski, 1997). While it has not been possible to determine the structure of the full-length factors, the structure of the archaeal WH domain from *Sulfolobus shibatae* has been determined by X-ray crystallography (Meinhart et al., 2003) and the structure of the ZR domain from human TFIIEα by NMR spectroscopy (Okuda et al., 2004). Recently, an archaeal homolog of the TFIIEβ subunit was identified in a subset of archaeal genomes, but nothing is known about its function (Blombach et al., 2009). In the absence of complete structural information about TFE, mechanistic insights into its role in transcription initiation come from a variety of biochemical experiments. TFE enhances promoter DNA melting during the formation of the RNAP-promoter open complex, possibly by interacting directly with the DNA nontemplate strand (NTS), and it preferentially binds to transcription initiation complexes formed on artificially melted “heteroduplex” promoter variants (Naji et al., 2007; Werner and Weinzierl, 2005). This is corroborated by biochemical evidence from the RNAPII system, where TFIIE can be crosslinked to the promoter DNA in the transcription bubble (Kim et al., 2000). Using a recombinant in vitro reconstituted RNAP system, we have shown that the activity of TFE crucially depends on the RNAP “stalk” consisting of subunits Rpo4/7 (Todone et al., 2001), which suggested a functional and possibly physical interaction between the RNAP stalk and TFE (Ouhammouch et al., 2004; Werner and Weinzierl, 2005). In order to explore proximities between transcription factors and RNAPII in the eukaryotic PIC, Hahn and coworkers derivatized yeast RNAP subunits with a photoactivatable crosslinker inserted in RPB1 and 2 (corresponding to Rpo1 and 2 in the archaeal annotation) and showed that TFIIE could be crosslinked to the RNAP clamp motif (Chen et al., 2007). However, this work could not provide information on a possible proximity between the RNAP stalk and TFIIE. The Rpo4/7 stalk promotes DNA melting at suboptimal temperatures (Naji et al., 2007) and plays a pivotal role during transcription elongation by enhancing processivity in vitro and in vivo (Hirtreiter et al., 2010a; Runner et al., 2008). In addition to RNAP subunits Rpo4/7, which suppress pausing (Hirtreiter et al., 2010b), several transcription elongation factors can release paused transcription elongation complexes, among them Spt4/5 (eukaryotes and archaea) and NusG (the bacterial homolog of Spt5). Not all NusG homologs have the same effect on RNAP, e.g., *T. thermophilus* NusG has been shown to reduce transcription elongation rather than increasing it (Sevostyanova and Artsimovitch, 2010). Spt4/5 and NusG associate with their cognate RNAPs by highly conserved interactions between the RNAP clamp coiled-coil motif and a hydrophobic depression in the Spt5 and NusG (NGN) domains (Hirtreiter et al., 2010a; Mooney et al., 2009b; Klein et al., 2011).

While the last couple of years have seen some new structural information on the architecture of transcription initiation complexes (Kostrewa et al., 2009; Liu et al., 2010), the position and conformation of TFIIF and TFIIE in the complexes has remained covert. Protein crosslinking combined with mass spectrometry has been used to obtain information about the interactions between RNAPII and TFIIF (Chen et al., 2010). For complexes where structural information is difficult to obtain from standard methodologies, measurement of fluorescence resonance energy transfer (FRET) followed by triangulation has proven to be successful (Mekler et al., 2002). An extension of this technique to the level of single molecules (Joo et al., 2008) allows us to obtain information about dynamic aspects (Margittai et al., 2003; Rasnik et al., 2004). Triangulation of single-molecule FRET (smFRET) distance information, combined with structural information and rigorous statistical analysis referred to as nanopositioning system (NPS), has been used to study the position of the exiting RNA (Andrecka et al., 2008), the influence of transcription factor TFIIB on the position of the nascent RNA (Muschielok et al., 2008), and the position of nontemplate and upstream DNA (Andrecka et al., 2009) in yeast RNAPII transcription elongation complexes.

Here we have used a recombinant in vitro transcription system based on the hyperthermophilic archaeon *Methanocaldococcus jannaschii* to investigate the structure and molecular mechanisms of the initiation and elongation factors TFE and Spt4/5, respectively. Using fluorescently labeled RNAP and TFE variants, we have applied the NPS to determine in solution the position of TFE in an archaeal preinitiation complex (PIC) consisting of RNAP, TBP, TFB, TFE, and promoter DNA. We find that the TFE WH domain binds to the RNAP clamp close to the clamp coiled-coil motif, and the TFE ZR domain binds at a position between the RNAP clamp and the RNAP stalk. Furthermore, using in-gel fluorescence quenching experiments, we have analyzed the spatial relationship between TFE domains and the DNA NTS. Since the binding site on RNAP for TFE identified in this work overlaps with the binding site on RNAP for Spt4/5 identified in previous work (Hirtreiter et al., 2010a), we carried out binding competition experiments and compared effects of TFE and Spt4/5 on RNAP activity during the initiation and elongation phases of transcription. We find that TFE and Spt4/5 compete for binding to RNAP and RNAP-containing complexes and that the relative binding affinities of TFE and Spt4/5 differ during initiation and elongation. During initiation, Spt4/5 can inhibit transcription, and TFE can efficiently displace Spt4/5 and overcome this inhibition. In contrast, during elongation, Spt4/5 efficiently displaces TFE. Our results identify the RNAP clamp as an important interaction site and regulatory hotspot for both initiation and elongation factors. They suggest that structural differences between RNAP in the PIC and TEC—e.g., in the clamp and/or in the position of the NTS—alter the affinity for TFE and Spt4/5 in a way that is important for the molecular mechanisms of transcription initiation, promoter escape, and transcription elongation.

## Results

### TFE Can Interact with Free RNAP, with RNAP in the PIC, and with RNAP in the TEC

**Figure 1:**
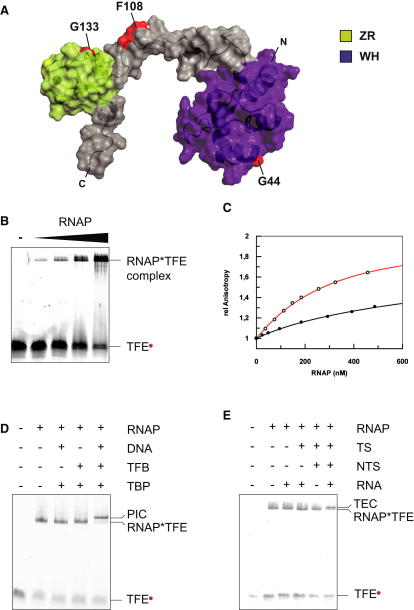
TFE Can Interact Directly with RNAP as Component of the Transcription Preinitiation Complex and the Ternary Elongation Complex (A) A homology model of TFE from *M. jannaschii*. The winged helix domain is highlighted in purple-blue, the ZR domain in lemon green. Fluorophore attachment sites are shown in red. (B) RNAP-TFE complexes; EMSA using TFE^133^∗^DL549^ (0.74 μM) and RNAP (0.2, 0.4, 1, and 2 μM). (C) Fluorescence anisotropy using labeled TFE^44^∗^Cy3B^ (50 nM) and wild-type RNAP (red curve) or RNAPΔRpo4/7 (black curve). Direct fitting of the titration curves yields a K_d_ of 0.2 ± 0.01 μM (wild-type RNAP) and 1.7 ± 0.15 μM (RNAPΔRpo4/7). (D) Complete PICs. EMSA using TFE^133^∗^DL549^ (0.74 μM), SSV T6 DNA (666 nM), TBP (8.7 μM), TFB (1 μM), and RNAP (1.2 μM). (E) TEC-TFE complexes. EMSA using TFE^133^∗^DL549^ (0.74 μM), TS DNA (15 μM), NTS DNA (20 μM), RNA (68 μM), and RNAP (1.2 μM).

In order to characterize the binding of TFE to RNAP, we produced fluorescently labeled TFE variants and carried out native gel electrophoresis experiments. The structure of *M. jannaschii* TFE has not been solved yet. In order to illustrate the size of the two principal TFE domains and to highlight the probe incorporation sites, we built a homology model (Experimental Procedures) using structural information on the WH (*Sulfolobus solfataricus* TFE, PDB: 1Q1H) and the ZR domains (*Homo sapiens* TFIIEα, PDB: 1VD4) (Figure 1A) and approximating the conformations of the interdomain linker and the C-terminal tail using minimum-energy considerations. The models of the WH and ZR domains show a good overall structural alignment with their parental structures (Figure S1). Recombinant TFE variants containing *p*-azido phenylalanine at positions 44 (WH domain), 108 (interdomain linker), and 133 (ZR domain) were produced, purified, and derivatized with the fluorescent probe DyLight 549 using Staudinger ligation (Chin et al., 2002) (Experimental Procedures). When labeled TFE was incubated with increasing amounts of RNAP, a species with lower electrophoretic mobility, corresponding to the RNAP-TFE complex, was formed in a concentration-dependent manner, indicating that TFE and RNAP can form a complex (Figure 1B). To confirm and quantify the interaction, we performed fluorescence-anisotropy experiments (Figure 1C). Upon addition of RNAP to fluorescently labeled TFE, fluorescence anisotropy increased in a concentration-dependent manner, with an apparent dissociation constant in the sub-μM range (K_d_ = 0.2 ± 0.01 μM). We next investigated the incorporation of TFE into the archaeal PIC. The PIC was assembled using SSV T6 promoter DNA oligonucleotides (Bell et al., 1999; Werner and Weinzierl, 2002), TBP, TFB, RNAP, and fluorescently labeled TFE. We utilized a promoter variant containing a 4 nucleotide (nt) heteroduplex region (−3/+1), which previously has been shown to form very stable PICs in the open complex conformation (Figure S4) (Werner and Weinzierl, 2005). In the presence of all components, a species with lower electrophoretic mobility than the RNAP-TFE complex was observed, corresponding to the complete archaeal PIC (Figure 1D). The assembly of the PIC was absolutely dependent on TBP and TFB. In order to test whether TFE also could associate with RNAP during the elongation phase of transcription, we assayed the binding of fluorescently labeled TFE to an archaeal TEC. RNAP can be recruited in a promoter-independent manner to synthetic elongation scaffolds consisting of a DNA template strand (TS), a nontemplate strand (NTS), and a 14 nt RNA oligomer to form a catalytically competent TEC (Hirtreiter et al., 2010a). We find that fluorescently labeled TFE can be recruited to the TEC, resulting in the formation of a species with slightly but unambiguously decreased electrophoretic mobility in a manner dependent on the TS, the NTS, and RNA (Figure 1E).

### The Location of TFE within the Archaeal PIC Complex

**Figure 2:**
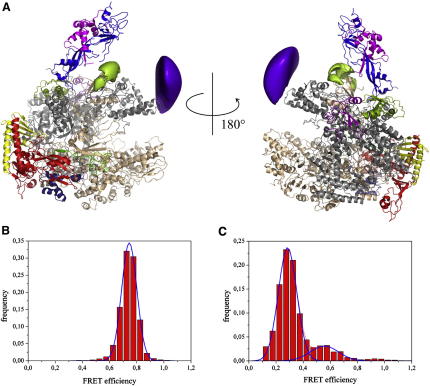
The Two TFE Domains Interact with Distinct Sites of the RNAP Clamp (A) Inferred locations of a fluorescent probe attached to residue 44 in the TFE WH domain (purple volume) and a fluorescent probe attached to residue 133 in the ZR domain (green volume). The size of each surface corresponds to 68% credible volumes. The X-ray structure of the archaeal polymerase of *S. solfataricus* (Hirata et al., 2008) (PDB: 2PMZ) is represented as ribbon, and each subunit is color-coded according to the convention. (B) Histogram of 898 sp-FRET trajectories for the FRET pair TFE-Rpo2″ (TFE^44APA^∗^Cy3B^ and Rpo2″^373APA^∗^DL649^). The single peak can be fitted with a Gaussian distribution that is centered at E = 0.74. (C) Histogram of 197 sp-FRET trajectories for the FRET pair TFE-Rpo7 (TFE^44APA^∗^Cy3B^ and Rpo7^S65C^∗^A647^), a main peak and a smaller side peak, which are fitted with Gaussian distributions centered at E = 0.29 and E = 0.56, respectively.

After we had established that TFE stably associates with RNAP, we sought to identify its precise binding site(s) on RNAP using NPS (Muschielok et al., 2008). In NPS, the location of a first entity (in this case TFE) relative to a second entity (in this case RNAP) is determined through the use of smFRET to obtain distance information for a fluorescent probe incorporated within the first entity and a set of complementary fluorescent probes incorporated at reference sites within the second entity. The use of Bayesian parameter estimation allows the computation of the most likely position and the three-dimensional uncertainty of the position of the fluorescent probe in the first entity (Figure S2). We incorporated a fluorescent probe at one site in each TFE domain (i.e., residue 44 in the TFE WH domain and residue 133 in the TFE ZR domain), and we incorporated a complementary fluorescent probe at each of five reference sites in RNAP (i.e., residue 257 of Rpo1′, residue 373 of Rpo2″, residue 11 of Rpo5, residue 49 of Rpo7, and residue 65 of Rpo7). Archaeal PICs were formed by incubating the SSV T6 promoter DNA oligonucleotides with TBP, TFB, TFE, and RNAP. For each single-molecule measurement, complexes having a fluorescence donor molecule attached to a TFE domain and a fluorescent acceptor attached to one of the five reference sites on RNAP were prepared. The complexes were immobilized and measured in a homebuilt TIRF microscope (Experimental Procedures). At least three smFRET measurements were performed for each pair of labeling sites. The FRET efficiency from all molecules was plotted as histograms and fitted with one or two Gaussian functions to extract the mean FRET efficiency. Corresponding histograms are shown in Figures 2B and 2C. All other histograms are shown in Figure S3, and the extracted data are summarized in Tables S1 and S2. For the NPS localization analysis of the position of the WH and the ZR domains of TFE in the PIC, first, the uncertainties due to the presence of flexible linkers between the probe and RNAP were computed (Figure S2), and the fluorescence anisotropies and the isotropic Förster radii were determined experimentally (Table S4). Three-dimensional probability densities were then calculated as in Andrecka et al., 2009 (Figure 2A and Table S3). The results indicate that the TFE WH domain interacts with RNAP in the PIC at or near the tip of the RNAP clamp coiled-coil motif (see purple volume in Figure 2A, denoting position of probe at TFE residue 44) and that the TFE ZR domain interacts with the RNAP within the PIC at or near the base of the RNAP clamp and the RNAP Rpo4/7 stalk (see green volume in Figure 2A, denoting position of probe at TFE residue 44). For each TFE domain, at least one smFRET histogram showed an additional minor subpopulation (≤20% of molecules) (Figures 2C and S3). No dynamic switching between the major and minor subpopulations was observed. We infer that each TFE domain may have an alternative, less favorable, but long-lived binding position. The NPS results indicate that, for each TFE domain, the inferred alternative binding position is immediately adjacent to the inferred primary binding position (Figure S6).

### The WH Domain of TFE Is Located Proximal to the Upstream Edge of the Transcription Bubble

**Figure 3:**
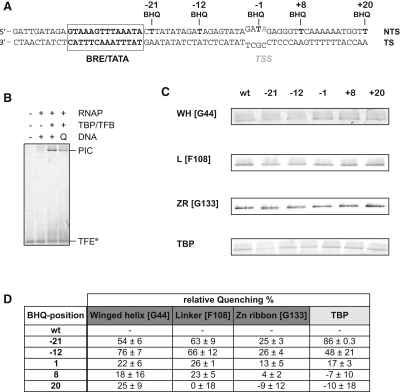
Fluorescence Quenching between TFE and NTS (A) Sequence of the SSV T6 promoter (transcription start site, TSS) and the location of BH quenchers. (B) PIC EMSA using TFE^DL549^ (246 nM), RNAP (1.2 μM), TBP (8.7 μM), TFB (1 μM), and DNA (667 nM). The quencher (Q) incorporated into the DNA nontemplate strand reduces fluorescence emission of fluorophores incorporated into TFE (shown for TFE^44^∗^DL549^). (C) PIC EMSAs (concentrations as in B) using individually labeled TFE domains (WH, winged helix; L, linker; ZR, Zinc ribbon) or labeled TBP (control). The promoter nontemplate strand DNA carried the BHQ-2 quencher molecule at positions −21, −12, −1, +8, or +20. (D) The fluorescence intensity of the PIC band was quantified and normalized to nonquenched wild-type (WT) PIC (based on at least three independent experiments).

In order to map the relative proximities of the two TFE domains and the interdomain linker to the NTS in the context of the PIC, we developed a fluorescence quenching assay by assembling PICs containing a fluorescence quencher (black hole quencher, BHQ-2) incorporated into the NTS at positions −21, −12, −1, +8, or +20 (Figure 3A). As in the above experiments, in order to ensure that the PIC was in the open complex conformation, we used a premelted heteroduplex promoter variant (Figure 3A). PICs were assembled with TFE fluorescently labeled at residue 44 (WH), 108 (linker), or 133 (ZR) and BHQ-2 derivatized or wild-type promoter DNA. The complexes were separated on native gels, and the PIC TFE fluorescence signal was quantitated in situ (Figures 3B–3D). For a positive control, we used fluorescently labeled TBP, which exhibited maximal quenching (86% quenching efficiency) when BHQ-2 was incorporated at position −21 just downstream of the TATA element (Figures 3 and S4). The TFE WH domain exhibited maximal quenching efficiency when BHQ-2 was incorporated at position −12 (76%), which is close to the upstream edge of the transcription bubble in the TEC (Andrecka et al., 2009). The TFE linker exhibited substantial quenching when BHQ-2 was incorporated at position −12 (66%) or position −21 (63%). The TFE ZR domain did not display substantial position-dependent differences in the fluorescence signal, suggesting that it is located approximately equidistant from the tested BHQ-2 incorporation positions in the NTS.

### The RNAP Clamp Coiled Coil and RNAP Stalk Are Required for TFE Binding and Activity

**Figure 4:**
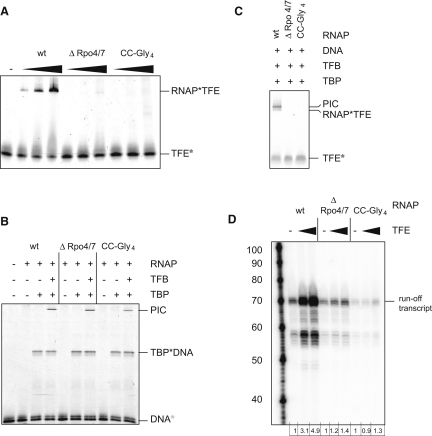
Mutations in the RNAP Clamp Coiled Coil and the Rpo4/7 Stalk Complex Interfere with TFE Recruitment and Activity (A) The RNAP-TFE complex; EMSA of TFE-RNAP complexes using TFE^133^∗^DL549^ (0.74 μM) and wild-type RNAP, RNAPΔRpo4/7, or CC-Gly4 (0.5, 1, and 2 μM). (B) PIC EMSA using fluorescently labeled DNA (Alexa 555) as tracer (67 nM), RNAP (1.2 μM), TBP (8.7 μM), and TFB (1 μM). (C) PIC EMSA using fluorescently labeled TFE (TFE^DL549^, 0.74 μM), RNAP (1.2 μM), TBP (8.7 μM), and TFB (1 μM). (D) Promoter-directed transcription assay using RNAP (1.2 μM), TBP (17.4 μM), TFB (2 μM), and TFE (0, 0.32, and 8 μM). The TFE stimulation is tabulated under the lanes.

In order to confirm the identified TFE domain binding sites, we made use of two previously described mutant variants of RNAP: a mutant in which ten residues of the tip of the RNAP clamp coiled-coil motif have been replaced by a tetra-glycine linker (the CC-Gly4 mutant) (Hirtreiter et al., 2010a) and a ten-subunit RNAP subassembly lacking Rpo4/7 (RNAPΔRpo4/7) (Hirtreiter et al., 2010a, 2010b; Ouhammouch et al., 2004; Werner and Weinzierl, 2005). In electrophoretic mobility shift assays (EMSAs), the addition of wild-type RNAP to fluorescently labeled TFE yielded a fluorescently labeled species with lower electrophoretic mobility, corresponding to the RNAP-TFE complex (Figure 4A). In contrast, the addition of the mutant variants RNAP CC-Gly4 and RNAPΔRpo4/7 failed to yield this species. We infer that the tip of the RNAP clamp coiled-coil motif and the Rpo4/7 stalk both are important for RNAP-TFE complex formation. Control experiments confirmed that both RNAP CC-Gly4 and ΔRpo4/7 are able to form stable PICs in a TBP/TFB-dependent fashion (Figure 4B). In order to quantify the contribution of the Rpo4/7 stalk to TFE binding, we repeated the fluorescence anisotropy experiments using RNAPΔRpo4/7 and found that the affinity for TFE was lower by approximately an order of magnitude (Figure 1C) (K_d_ = 1.7 ± 0.15 μM). We conclude that the Rpo4/7 complex is important for the binding of TFE to RNAP. We infer that the Rpo4/7 complex physically interacts with TFE, in agreement with the NPS localization of the ZR domain described above, and/or allosterically affects the conformation of the binding site for TFE. We directly observed TFE recruitment to PIC using fluorescently labeled TFE in EMSAs. Neither RNAP mutant variant was able to recruit TFE into the PIC (Figure 4C). In order to monitor the impact of TFE on transcription initiation, we developed a promoter-directed transcription runoff assay using the SSV T6 promoter. In the presence of TBP and TFB, RNAP initiates start-site-specific transcription from this strong viral promoter. The linearized plasmid template directs the synthesis of a 70 nt runoff transcript (Figure 4D). The addition of increasing amounts of TFE stimulates transcription without qualitatively altering the transcript pattern (Figure 4D). The TFE binding-deficient RNAP variants RNAP CC-Gly4 and RNAPΔRpo4/7 were able to synthesize the runoff transcript, albeit at reduced levels (Figure 4D). However, while transcription by the wild-type RNAP was stimulated by TFE about 5-fold, neither of the mutant variants was able to respond to TFE to an extent comparable to the wild-type RNAP (Figure 4D).

### The Elongation Factor Spt4/5 Can Inhibit PIC Formation and Transcription Initiation

**Figure 5:**
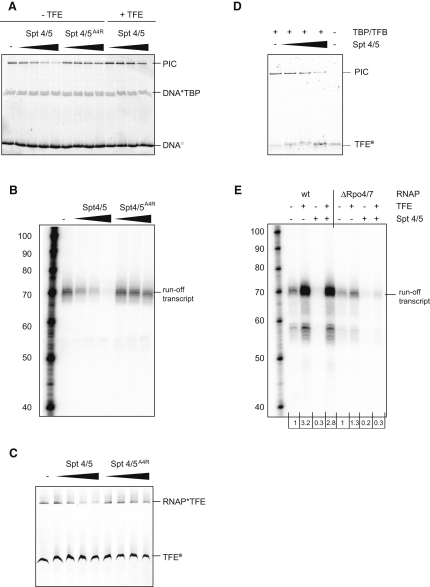
Spt4/5 and TFE Compete for RNAP Binding during Transcription Initiation, and TFE Alleviates the Repression of Spt4/5 (A) The PIC complex is destabilized by Spt4/5 and rescued by TFE. Fluorescently labeled SSV T6 promoter DNA (67 nM) was incubated with 1.2 μM RNAP, 8.7 μM TBP, and 1 μM TFB in the presence or absence of TFE (8 μM) and increasing amounts of WT Spt4/5 or the RNAP binding-deficient mutant Spt4/5^A4R^ (5, 18, 60, and 147 μM). (B) Spt4/5 represses promoter-directed transcription in the absence of TFE. Reactions included RNAP (1.2 μM), TBP (17.4 μM), TFB (2 μM), TFE (0, 0.32, and 8 μM), and Spt4/5 or Spt4/5^A4R^ (5, 18, and 55 μM). (C) Spt4/5 displaces RNAP-bound TFE. Increasing amounts of Spt4/5 or Spt4/5^A4R^ (0.33, 1, 7.5, and 25 μM) were added to a preformed RNAP^∗^TFE^133^∗^DL549^ (0.74 μM) complex. (D) Addition of increasing amounts of WT Spt4/5 (0, 16, 32, and 137 μM) to preformed PICs using fluorescently labeled TFE (0.75 μM). (E) Promoter-directed transcription using either WT RNAP or RNAPΔRpo4/7 (1.2 μM), TFE (8 μM), and Spt4/5 (55.2 μM). The effect of Spt4/5 and TFE on transcription is tabulated under the lanes.

Spt4/5 stimulates the processivity of RNAP (Hirtreiter et al., 2010a), and while the molecular mechanisms are still not completely understood, it is believed that Spt4/5 modulates the DNA binding properties of RNAP (Grohmann and Werner, 2010). We tested the influence of Spt4/5 on the recruitment of RNAP to the PIC in EMSAs using fluorescently labeled DNA, TBP, and TFB. Interestingly, the addition of Spt4/5 prevented the formation of the minimal PIC in a dose-dependent manner (Figure 5A). The effect was specific. Thus, a mutant variant of Spt4/5 carrying a single substitution (A4R) in the Spt5 NGN domain that abrogates RNAP binding failed to exhibit this activity (Figure 5A) (Hirtreiter et al., 2010a). In order to determine whether this activity was also reflected in transcription initiation, we carried out promoter-directed runoff transcription assays. Consistent with the results of the recruitment experiments, the results of the transcription assays show that the addition of Spt4/5 to minimal transcription complexes consisting of DNA, TBP, TFB, and RNAP inhibited transcription (Figure 5B) (IC_50_ = 9.6 ± 5 μM) and that Spt4/5-A4R had no effect.

### TFE Efficiently Prevents Inhibition of Transcription Initiation by Spt4/5

Our NPS results (Figure 2A) and our molecular genetics results with the RNAP CC-Gly4 mutant (Figure 4A) indicate that the binding site of the TFE maps to the same part of RNAP that previously has been shown to serve as the binding site for the Spt5 NGN domain, i.e., the tip of the RNAP clamp coiled-coil motif (Hirtreiter et al., 2010a). To determine whether the binding sites for TFE and Spt4/5 overlap, we performed binding competition experiments using fluorescently labeled RNAP-TFE complexes. The addition of Spt4/5 prevented the formation of RNAP-TFE complexes in a concentration-dependent fashion, indicating that Spt4/5 and TFE compete for binding to RNAP (Figure 5C). The RNAP binding-deficient mutant variant Spt4/5 A4R had no effect on the RNAP-TFE complexes (Figure 5C). The IC_50_ of Spt4/5 for the negative effect on the RNAP-TFE complex was 0.55 ± 0.14 μM (Figure S5).

We subsequently investigated the combined effects of TFE and Spt4/5 on formation of the PIC. We assessed effects of Spt4/5 on the formation of the complete PIC (RNAP, TBP, TFB, TFE, and promoter DNA) in EMSAs using fluorescently labeled DNA as tracer and found that the presence of TFE prevented the inhibition of PIC formation by Spt4/5, reducing inhibition to the level observed with the mutant variant Spt4/5 A4R (Figure 5A). We repeated the PIC EMSAs using fluorescently labeled TFE as tracer in order to test whether Spt4/5 could displace TFE from the PIC (Figure 5D). We found that Spt4/5 could displace TFE from the PIC (Figure 5D), but that it could do so only very inefficiently, requiring a 50-fold higher concentration to displace TFE from the PIC than to displace TFE from RNAP-TFE (IC_50_ = 29 ± 17 μM versus IC_50_ = 0.55 ± 0.14 μM) (Figures 5C and S5). We analyzed whether TFE could prevent the inhibition of transcription initiation by Spt4/5. The addition of TFE to minimal transcription reactions (DNA, TBP, TFB, and RNAP) increased the transcript synthesis by approximately 5-fold, in agreement with previous observations (Bell et al., 2001; Naji et al., 2007; Werner and Weinzierl, 2005). In contrast, the addition of Spt4/5 inhibited transcript synthesis by more than 10-fold (Figure 5E). The addition of TFE prevented the Spt4/5-dependent inhibition of transcription initiation by Spt4/5, leading to transcript levels identical to those in reactions in which Spt4/5 was omitted (Figure 5E). For a negative control, we used the RNAPΔRpo4/7 variant, which is defective in TFE binding (Figure 3A). Under these conditions TFE stimulated transcription less than 1.2-fold, Spt4/5 repressed transcription similarly to the wild-type RNAP, and TFE only marginally compensated for this repression (Figure 5E).

### Spt4/5 Displaces TFE from the TEC

**Figure 6:**
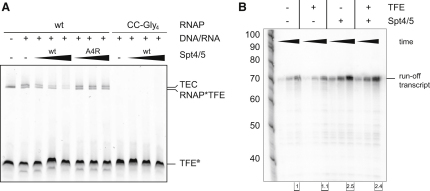
Spt4/5 Displaces TFE from Transcription Elongation Complexes (A) Spt4/5 efficiently competes for TFE binding in the TEC. EMSAs were conducted using WT RNAP or RNAP CC-Gly4 (0.21 μM), TEC (NTS, 20 μM; TS, 15 μM; RNA, 68 μM), fluorescently labeled TFE^G133^∗^DL549^ (0.74 μM), and increasing amounts of Spt4/5 or Spt4/5^A4R^ (1, 2.5, and 15 μM). (B) Transcription elongation assay using WT RNAP (420 nM), TFE (2.5 μM), and Spt4/5 (10 μM). Spt4/5 stimulates elongation in the presence of TFE. Reactions were stopped at 1.5, 3, and 10 min. The runoff transcript levels were quantified and are indicated under the lanes.

Our data showed that relative affinities of TFE and Spt4/5 to RNAP are context dependent: Spt4/5 efficiently displaces TFE from the RNAP-TFE complex (IC_50_ = 0.55 ± 0.14 μM), but only inefficiently displaces TFE from the PIC (IC_50_ = 29 ± 17 μM). In order to test the binding characteristics of TFE and Spt4/5 during transcription elongation, we carried out binding and transcription assays using synthetic elongation scaffolds consisting of DNA TS, NTS, and a short RNA primer (RNA). As observed previously, TFE forms a complex with RNAP (Figure 6A). In the presence of TS, NTS, and RNA, a species with lower electrophoretic mobility than that of RNAP-TFE appears, corresponding to the TEC-TFE complex (Figure 6A). The addition of increasing amounts of Spt4/5 efficiently prevented the formation of the TEC-TFE complex, with a half-maximal inhibitory concentration comparable to the RNAP-TFE complex: IC_50_ = 0.79 ± 0.07 μM. For negative controls, we made use of the RNAP binding-deficient Spt4/5 A4R mutant, which had no effect on the TEC-TFE complex (Figure 6A). We complemented the binding studies with transcription elongation assays using synthetic elongation scaffolds. RNAP can be recruited factor-independently to the scaffolds and upon NTP addition extends the 14-mer RNA primer to form a 72 nt runoff transcript (Figure 6B). Whereas the addition of TFE has no substantial effect on elongation, the addition of Spt4/5 stimulates the synthesis of the runoff transcript, as observed previously (Figure 6B) (Hirtreiter et al., 2010a). Importantly, the Spt4/5 stimulation was not significantly reduced by the addition of TFE, indicating that Spt4/5 remains associated with the TEC in the presence of TFE (Figure 6B).

## Discussion

The FRET and mutational analyses presented here identify two discrete positions on the RNAP clamp as the binding site for TFE: the TFE WH domain interacts with the tip of the RNAP clamp coiled-coil motif (subunit Rpo1′), and the TFE ZR domain interacts with base of the RNAP clamp (Rpo1′ and Rpo2″) and is in close proximity to the RNAP stalk (Rpo4/7) (Figure 2A). These binding sites provide a framework for understanding published results on TFE and, by inference, TFIIE. First, both TFE and TFB interact with the RNAP clamp coiled coil and possibly with each other, which may account for why TFE can complement mutations in the TFB linker region in RNAP recruitment and transcription assays (Werner and Weinzierl, 2005). Second, the proximity of the WH domain and the NTS at the upstream edge of the transcription bubble (Figure 3) accounts for the reported crosslinking between TFE and the NTS (Grünberg et al., 2007) and between eukaryotic TFIIE and promoter DNA in the transcription bubble (Kim et al., 2000). Third, the proximity between the TFE ZR domain and the RNAP stalk provide a rationale for the Rpo4/7 dependency of TFE activity (Naji et al., 2007; Werner and Weinzierl, 2005). The two binding sites on the archaeal RNAP for the TFE WH and ZR domains are in excellent agreement with the results obtained in the eukaryotic RNAPII system by Hahn and coworkers (Chen et al., 2007) and recent studies in the RNAPIII system. Yeast RNAPII subunits Rpb1 and 2 were derivatized with crosslinkers, and eukaryotic TFIIE could be crosslinked to residues RPB1 His213 and 286 (corresponding to Lys186 and Gln259 in *S. solfataricus*) after formation of the PIC. Both residues reside in the RNAP clamp domain and are proximal to the location of the TFE WH domain identified with NPS (Figure S6). In the RNAPIII system, a subcomplex of subunits C82/C34/C31 (C82/C34 are homologs of TFIIEα and TFIIEβ) is stably associated with the RNAPIII core and essential for transcription initiation. A comparison between the crystal structure of yeast RNAPII and the cryo-EM surface envelope of RNAPIII has allowed the identification of additional densities that have been assigned to RNAPIII-specific subunits (Fernández-Tornero et al., 2007; Lefèvre et al., 2011). In congruence to our NPS data, both the C82/C34/C31 subcomplex as well as hRPC62 (a human ortholog of C82) have been assigned to densities next to the clamp and the stalk.

**Figure 7:**
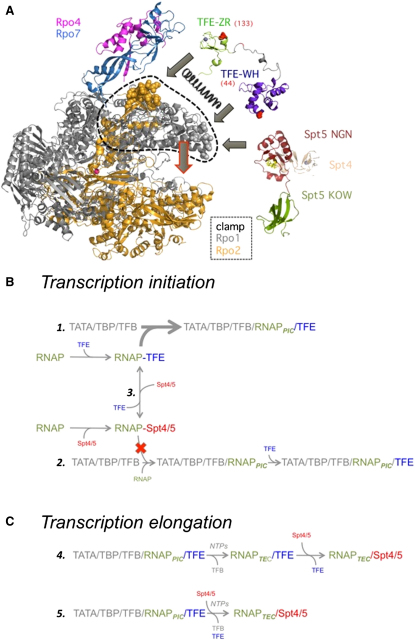
Molecular Mechanisms of TFE during Open Complex Formation (A) The TFE WH (highlighted in green) and ZR domains (purple blue) interact with the RNAP clamp on the tip of the Rpo1 coiled coil (gray spheres) and with Rpo2 (orange spheres) at the base of the Rpo4/7 stalk, respectively. The Spt5 NGN domain (red) interacts with the clamp coiled coil (gray spheres). RNAP structure representation is based on *S. shibatae* (PDB: 2WAQ), and TFE is a homology model of *M. jannaschii* TFE (Experimental Procedures). Rpo2 is highlighted in orange, Rpo4 in magenta, Rpo7 in sky blue, and Rpo1 and all other RNAP subunits in gray. We envisage that the bidentate RNAP-TFE interaction mode (indicated with gray block arrows) provides the necessary purchase for TFE to close/open the RNAP clamp (dashed black circle) and thereby alter the width of the DNA binding channel (red block arrow). The movement of the clamp (indicated with a spring) is likely to play an important role during DNA melting and the loading of the template strand into the active site. (B) Recruitment pathways during transcription elongation. The TATA/TBP/TFB platform can recruit the RNAP-TFE complex (1) or first RNAP and subsequently TFE (2) to form the preinitiation complex (PIC). Free RNAPs can associate with TFE or Spt4/5. The RNAP-Spt4/5 complex is barred from efficient recruitment (red cross) to the TATA-TBP-TFB platform, but TFE overcomes this impediment by displacing RNAP-bound Spt4/5 (3) to form RNAP-TFE complexes that are readily recruited to the promoter. (C) Recruitment of Spt4/5 during transcription elongation. Following promoter escape, TFE can remain associated with RNAP, forming a TEC-TFE complex. Spt4/5 can efficiently displace TFE from the TEC-TFE complex and stimulate processivity (4). Alternatively, Spt4/5 engages with the PIC at the transition of transcription initiation and elongation during promoter escape (5).

Our NPS data have enabled us to position the two individual WH and ZR domains of archaeal TFE on discrete parts of the clamp motif. These two binding sites provide the basis to suggest specific structural hypotheses for the mechanism of action of TFE and, by inference, TFIIE. First, our results suggest that the WH and ZR domains both interact with the RNAP clamp. In principle, contacts of the two TFE domains with two different sites on the RNAP clamp might help “prise” the RNAP clamp into a specific open or closed conformation (Figure 7A). Second, our results suggest that the TFE ZR domain interacts with the base of the RNAP clamp close to the RNAP stalk. In principle, the TFE ZR domain could “wedge” between the RNAP clamp and the remainder of RNAP and/or between the RNAP clamp and the RNAP stalk, helping to “lock” the RNAP clamp in a specific open or closed conformation. By either of the above two hypotheses, TFE would induce or stabilize a conformational change that would affect the width of the DNA binding channel and thereby would affect loading of the template DNA. Our results also suggest that the TFE WH domain interacts with the NTS at the upstream edge of the transcription bubble. In principle, interactions of TFE with the NTS could help favor promoter melting and open complex formation.

The eukaryotic and archaeal transcription elongation factor Spt4/5 and its bacterial counterpart NusG previously have been shown to interact with the tip of the RNAP clamp coiled-coil motif and to stimulate transcription elongation (Hirtreiter et al., 2010a). Here, we show that Spt4/5 additionally has an opposite effect on transcription initiation: Spt4/5 inhibits PIC formation and transcription initiation. Since the Spt4/5 (NusG) binding site is located on the tip of the RNAP clamp and is close to the RNAP DNA binding channel, it is likely that Spt4/5 (NusG) modulates the interaction of RNAP with the DNA and/or with the DNA-RNA hybrid (Grohmann and Werner, 2010). In principle, Spt4/5 (NusG) might allosterically favor closed conformational states of the RNAP clamp, thereby indirectly interfering with entry of DNA into the RNAP DNA binding channel and/or departure of DNA from the RNAP DNA binding channel. Our results provide two additional lines of support for this hypothesis. First, Spt4/5 inhibits formation of the PIC and inhibits promoter-dependent transcription initiation. Second, Spt4/5 stimulates transcription elongation in assays that do not involve promoter-dependent transcription initiation but instead utilize linear DNA-RNA scaffolds. A low-resolution cryo-EM structure of the RNAP-Spt4/5 complex and a model based on an X-ray structure of a recombinant clamp-Spt4/5NGN complex (both from *Pyrococcus furiosus*) confirm our results (Klein et al., 2011; Martinez-Rucobo et al., 2011). A density corresponding to the Spt4/5NGN core closes the gap across the DNA binding channel; it could prevent entry to and release of template DNA from the RNAP.

We discovered that TFE is able to overcome the inhibitory effects exerted by Spt4/5 during transcription initiation by virtue of competitive displacement of Spt4/5. Figure 7 illustrates a working model of TFE and Spt4/5 action during transcription initiation and elongation. In our experiments, TFE prevails over Spt4/5 in the competition for binding to RNAP in the context of PIC (possibly due to contacts between TFE and TFB and/or possibly due to PIC-specific contacts between TFE and RNAP and/or between TFE and the NTS). Once RNAP has escaped the promoter, the relative affinities of Spt4/5 and TFE are reversed: in the context of the TEC, Spt4/5 prevails over TFE in the competition of binding to RNAP (Figure 7C). It is also possible that Spt4/5 displaces TFE from the PIC earlier, during promoter escape, and thus stimulates transcription at the transition between initiation and elongation (Figure 7C). Our data thus provide in vitro evidence for a mechanism of transcription initiation and elongation factors that compete for RNAP binding. The RNAP clamp coiled-coil motif is a conserved binding site for the initiation factors TFB and TFE (eukaryotes and archaea) and sigma70 (bacteria) and for the elongation factors Spt4/5 (eukaryotes and archaea) and NusG (bacteria) (Belogurov et al., 2007; Hirtreiter et al., 2010a; Kostrewa et al., 2009). Moreover, NusG and its paralog RfaH compete with sigma70 for binding to RNAP (Sevostyanova et al., 2008), highly reminiscent of Spt4/5 and TFE in the archaea and, by inference, in eukaryotes. NusG has pleiotropic effects on elongation; it is a positive elongation factor that increases processivity but enhances transcription termination in the context of rho (Mooney et al., 2009a). Similarly, Spt4/5 may modulate transcription in more than one way. Spt4/5 stimulates processivity (Hirtreiter et al., 2010a), and our results presented here demonstrate that it inhibits transcription initiation. The eukaryotic Spt4/5 complex has multiple KOW domains and C-terminal repeat regions and interacts with a plethora of factors involved in chromatin remodeling, RNA processing, and polyA site selection (Cui and Denis, 2003; Lindstrom et al., 2003; Schneider et al., 2006). In summary, Spt5-like transcription factors are not only universally conserved in evolution, but also highly versatile.

## Experimental Procedures

### Recombinant Protein Production and Labeling

Unlabeled transcription factors TBP, TFB, TFE, and Spt4/5 were produced as described previously (Hirtreiter et al., 2010a; Werner and Weinzierl, 2005). Recombinant RNAP was reconstituted as described previously (Werner and Weinzierl, 2002). Rpo5 and 7 were labeled with fluorescent probes as described previously (Grohmann et al., 2009). Rpo1′, Rpo2″, and TFE were labeled using a nonsense suppressor strategy (Chin et al., 2002) (Supplemental Information).

### Comparative Modeling

The TFE WH domain was modeled based on the *S. solfataricus* TFE N-terminal domain crystal structure (PDB: 1Q1H, resolution 2.9 Å) (Meinhart et al., 2003), and the TFE ZR domain was modeled based on the human TFIIEα NMR structure ensemble (PDB: 1VD4) (Okuda et al., 2004), both using Modeler 9.7 (build 6923) (Sali and Blundell, 1993). Stereochemistry was checked using Procheck V3.4 (Laskowski et al., 1993). The TM score for the WH domain model is 0.93 and the average rmsd 1.24 Å, and for the ZR domain model 0.62 and 2.09 Å, respectively (Figure S1). The two domains were connected by an initially coiled linker of 12 aa missing in the templates and energy minimized in a 3 ns unconstrained molecular dynamics simulation (in explicit water with ions) using simulated annealing energy minimization with the force field Amber99 (Wang et al., 2000) as implemented in Yasara (Krieger et al., 2002).

### Fluorescence Anisotropy

Fluorescence anisotropy of labeled TFE and TFE-RNAP complexes was recorded as previously described (Grohmann et al., 2009).

### PIC Preparation and NPS Experiments

Nucleic acid scaffolds were used to assemble preinitiation complexes consisting of a 65 nt long double-stranded DNA with template and nontemplate DNA strands containing a 4 bp mismatch around the active site (m3 template [Werner and Weinzierl, 2005]). For surface immobilization of the complexes, the nontemplate DNA strand had Biotin attached at the 5′ end via a C6-amino linker. The DNA strands were purchased from IBA (Göttingen, Germany). The DNA strands were annealed as described before (Andrecka et al., 2008). The PIC complexes were assembled by adding 1 μl each of nucleic acid scaffold (2 μM), TBP (10 μM), TFB (10 μM), RNAPΔRpo4/7 (2 μM), and Rpo4/7 (10 μM) to 10 μl HMNE buffer (40 mM HEPES [pH 7.3], 250 mM sodium chloride, 2.5 mM magnesium chloride, 0.1 mM EDTA, 5% glycerol and 10 mM dithiothreitol). The mixture was then incubated at 55°C for 10 min, and complete PIC complexes were purified using Microcon-YM100 centrifugal filters (Millipore) against HMNE buffer. Then 1 μl TFE (12.4 μM) was added to the purified complexes and incubated for 10 min at 55°C.

NPS was carried out as described previously (Andrecka et al., 2008, 2009; Muschielok et al., 2008). For a detailed description of NPS setup and calculations, refer to the Supplemental Information.

### Electrophoretic Mobility Shift Assays

The reaction components indicated in the figure legends were combined on ice in 1× HNME buffer, incubated for 20 min at 65°C, and separated on 10%–12% native Tris-glycine gels or 4%–12% Tris-glycine gradient gels (Bio-Rad and Invitrogen) at 200 V for 45 min at room temperature (Werner and Weinzierl, 2005). For PIC promoter templates and synthetic elongation scaffolds, complementary DNA strands and RNA were annealed by incubation for 5 min at 95°C and slowly cooled down to room temperature. The final concentrations were as follows: TFE, 740 nM; RNAP, 1.2 μM; TBP, 8.7 μM; TFB, 1 μM; TS, 667 nM; NTS, 667 nM; Heparin, 6.7 μg/ml. Fluorescently labeled TFE and TFE-containing complexes were visualized on a Fuji FLA2000 scanner, and signals were quantified using Image Gauge software (Fuji Science Lab 2003).

### Transcription Assays

Promoter-directed transcription runoff assays were carried out by combining 666 nM RNAP, 17.5 μM TBP, and 2 μM TFB with 1.5 μg pGEM-SSV T6 linearized with NcoI in a total volume of 15 μl (Werner and Weinzierl, 2002). All components were combined on ice, and transcription was initiated by the addition of 0.75 mM ATP, UTP, and GTP substrates containing 2 μM CTP and 75 pM [α-^32^P]CTP (0.3 μl of 3000 Ci/mmol, Perkin Elmer). Ten microliters of the reactions were stopped by the addition of 15 μl formamide loading buffer. The ^32^P-labeled fragments were separated on 10% urea PAGE for 80 min at 80 W and visualized using a Fuji FLA2000 scanner, and the signals were quantified using Image Gauge software (Fuji Science Lab). Transcription elongation assays using synthetic elongation scaffolds were carried out as previously described (Hirtreiter et al., 2010b).

## Supplemental Information

Document S1. Seven Figures, Four Tables, Supplemental Experimental Procedures, and Supplemental References
