# Supplementary material for: The Initiation Factor TFE and the Elongation Factor Spt4/5 Compete for the RNAP Clamp during Transcription Initiation and Elongation
Source: Mol Cell. 2011 Jul 22;43(2-2):263–74. doi: 10.1016/j.molcel.2011.05.030 (PMC3223566; doi:10.1016/j.molcel.2011.05.030)
Supplement: Document S1. Seven Figures, Four Tables, Supplemental Experimental Procedures, and Supplemental References [file mmc1.pdf]

Supplemental Information

Molecular Cell, *Volume 43*

The Initiation Factor TFE and the Elongation Factor Spt4/5 Compete for Binding to the RNAP Clamp during Transcription Initiation and Elongation

Dina Grohmann, Julia Nagy, Anirban Chakraborty, Daniel Klose, Daniel Fielden, Richard H. Ebright, Jens Michaelis, and Finn Werner

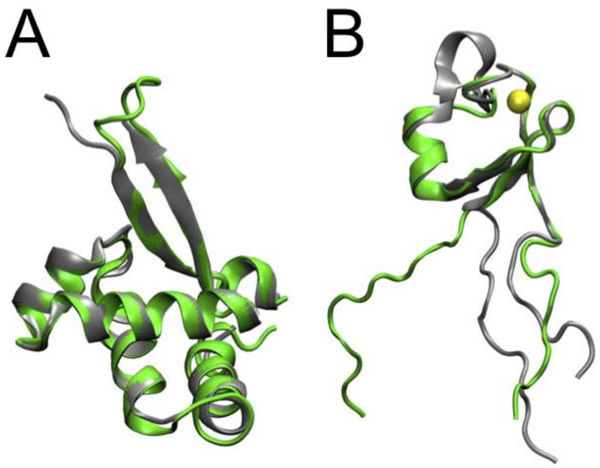

Figure S1. Homology Model of *M. jannaschii* TFE

(A and B) Structural alignment of the models (coloured in green) of the *M. jannaschii* TFE WH- (A) and ZR-domains (B) with the structures (coloured in grey) of the *S. solfataricus* WH- (pdb 1Q1H, A) and the *H. sapiens* TFIIE alpha ZR-domains (1VD4, B). Structural alignments were made using VMD (Humphrey et al., 1996). The Zn-ion is highlighted as yellow sphere.

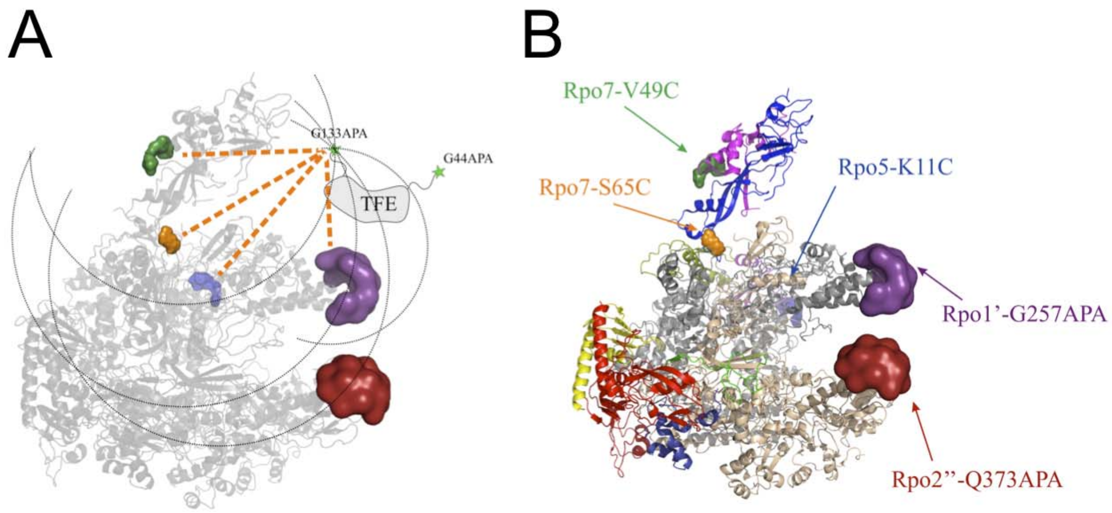

Figure S2. The NPS System

(A) We have used NPS to determine the position of the winged helix domain and the zinc-ribbon domain of TFE in the PIC (A). To this end, smFRET efficiencies between a fluorescent probe attached to either WH- or ZR-domain of TFE (green star), and fluorescent probes attached at reference sites in RNAP (colored volumes) were measured, providing information about their respective distances (orange dashed lines). From these distances the ADM position can be computed by trilateration (indicated by black dashed lines) in a technique called Nano Positioning System (NPS) (Muschielok et al., 2008). (B) Modelled locations of reference fluorescent probes used in NPS localisation of TFE. Calculated satellite position priors of subunit Rpo7-V49 (green), Rpo7-S65 (orange), Rpo5-K11 (blue), Rpo1'-G257 (purple) and Rpo2''-Q373 (red) are shown together with the X-ray structure of the archaeal polymerase of *S. solfataricus* (pdb file: 2pmz) (Hirata et al., 2008). This figure was prepared using PyMOL.

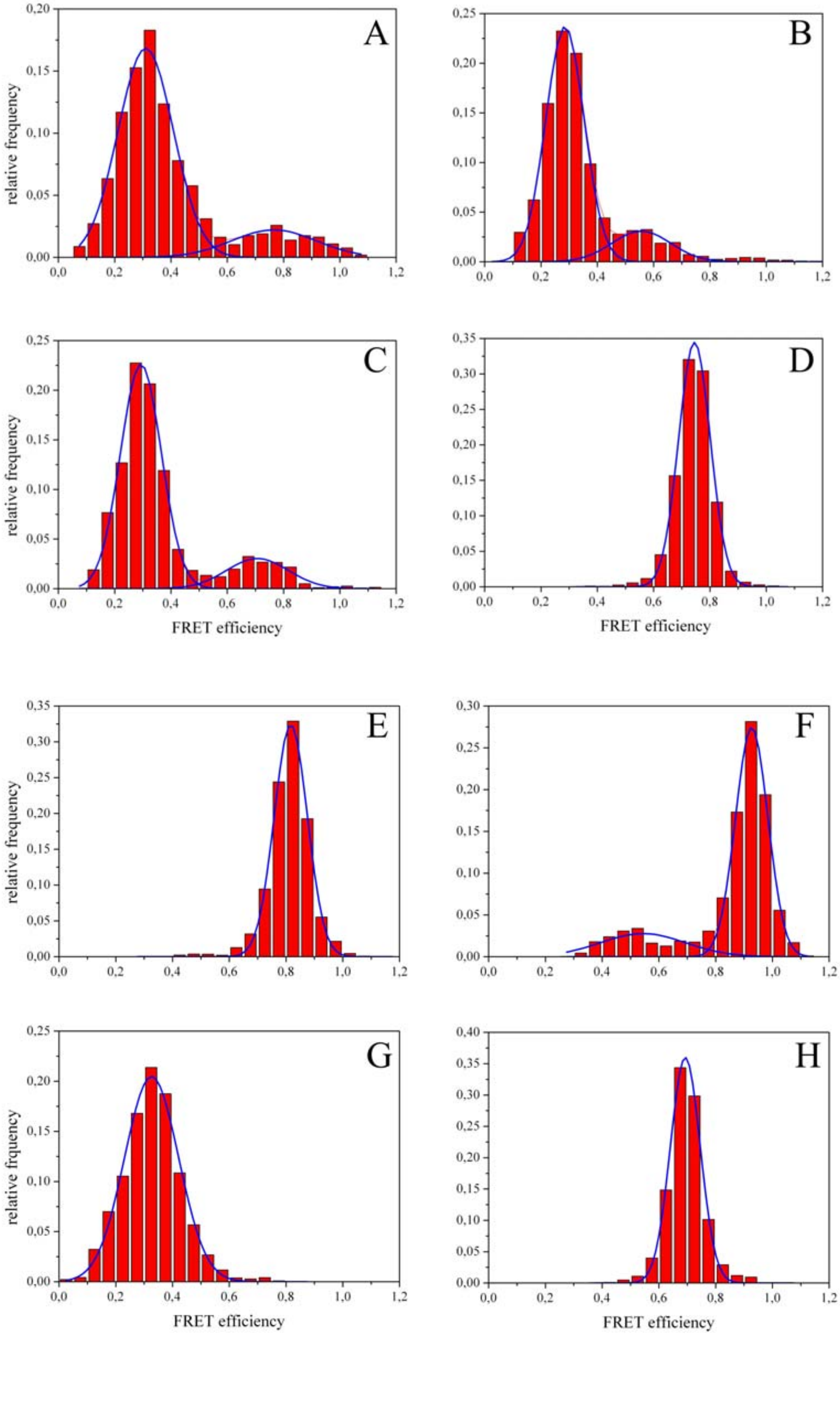
A
B
C
D
E
F
G
H
relative frequency
relative frequency
relative frequency
relative frquency
FRET efficiency
FRET efficiency
FRET efficiency
FRET efficiency

Figure S3. Complete Histograms of the Measured FRET Pairs Used to Determine the Position of the Winged Helix Domain (A-D) and the Zinc-Ribbon Domain (E-H) of TFE

Shown are the frame-wise histograms of (A) TFE^{G44} – Rpo7-V49; (B) TFE^{G44} – Rpo7-S65; (C) TFE^{G44}– Rpo5-K11; (D) TFE^{G44} – Rpo2''-Q373; (E) TFE^{G133} – Rpo7-V49; (F) TFE^{G133} – Rpo7-S65; (G) TFE^{G133} – Rpo2''-Q373; (H) TFE^{G133} – Rpo1'-G257. The histograms where fitted with a double (A-C, F) or single (D-E, G-H) Gaussian distribution indicated by the blue lines (all results of the fits are summarized in Table S1 and S2).

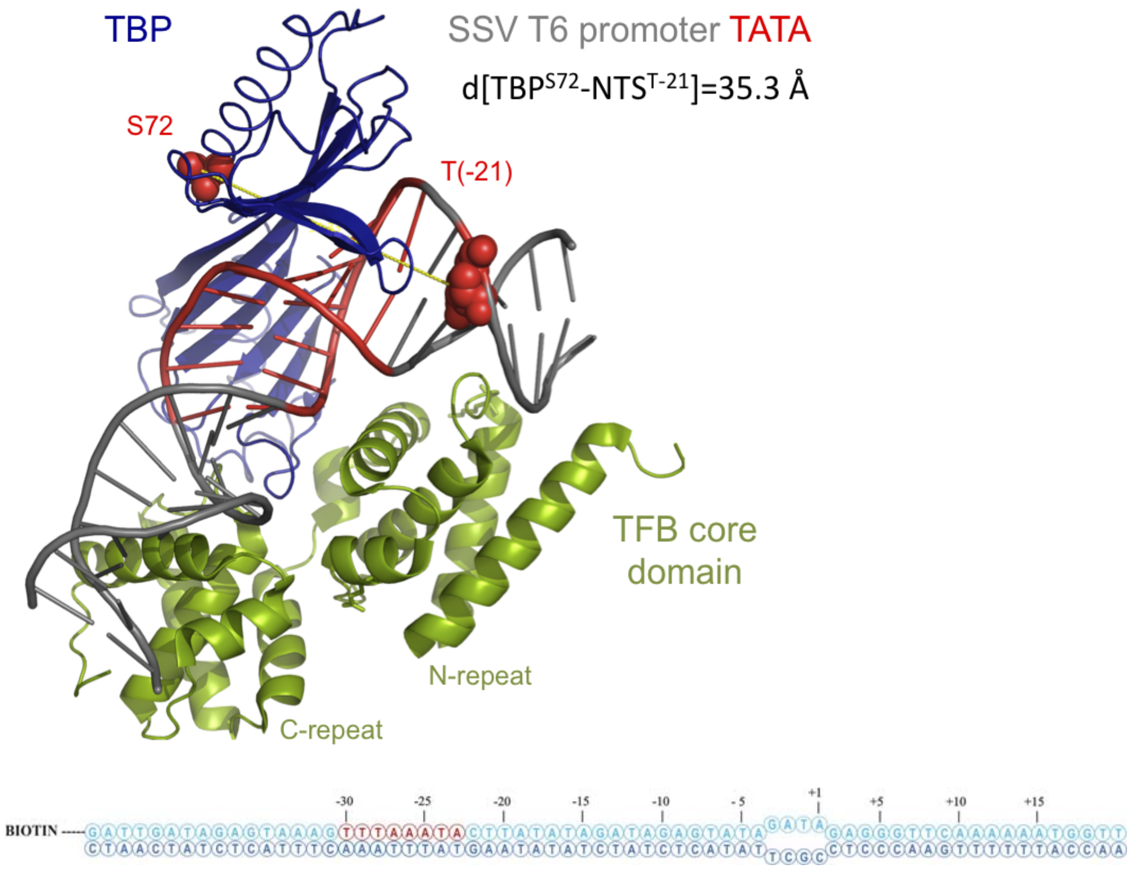

Figure S4. Structure of the TBP-TFB$_{core}$-SSVT6 Promoter Complex

In order to measure fluorescence quenching between TBP and the nontemplate strand (NTS) at position -21 we assembled preinitiation complexes with fluorescently labelled TBP (S72C-Alexa555, highlighted with red spheres), TFB, TFE, RNAP and a SSV T6 promoter oligonucleotides containing the black hole quencher BHQ-2 at position -21 (highlighted with red spheres). The distance between TBP^{S72} and NTS$^{T(-21)\ in}$ in the TBP/TFB$_{core}$-TATA complex is 35.3 Å (TATA consensus sequence is highlighted in red). The structure in the figure is *Pyrococcus furiosus* TBP (blue) and the TFB core domain (green, encompassing the N- and C-terminal cyclin repeats) bound to the viral SSV T6 promoter, which is used throughout this study (Littlefield et al., 1999). The template includes a short heteroduplex region (-3/+1) that stabilised the PIC by forming the open complex. The TATA box is highlighted in red.

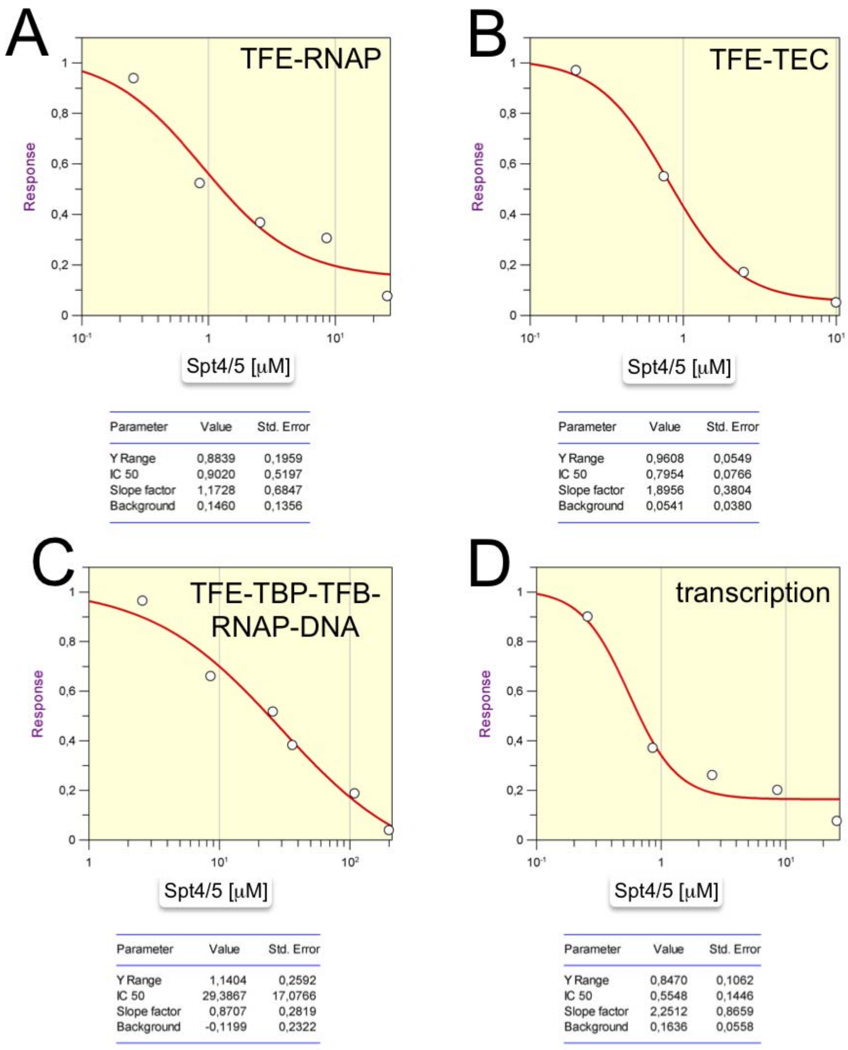

A

Parameter	Value	Std. Error
Y Range	0,8839	0,1959
IC 50	0,9020	0,5197
Slope factor	1,1728	0,6847
Background	0,1460	0,1356

B

Parameter	Value	Std. Error
Y Range	0,9608	0,0549
IC 50	0,7954	0,0766
Slope factor	1,8956	0,3804
Background	0,0541	0,0380

C

Parameter	Value	Std. Error
Y Range	1,1404	0,2592
IC 50	29,3867	17,0766
Slope factor	0,8707	0,2819
Background	-0,1199	0,2322

D

Parameter	Value	Std. Error
Y Range	0,8470	0,1062
IC 50	0,5548	0,1446
Slope factor	2,2512	0,8659
Background	0,1636	0,0558

Figure S5. Competition between Spt4/5 and TFE for RNAP

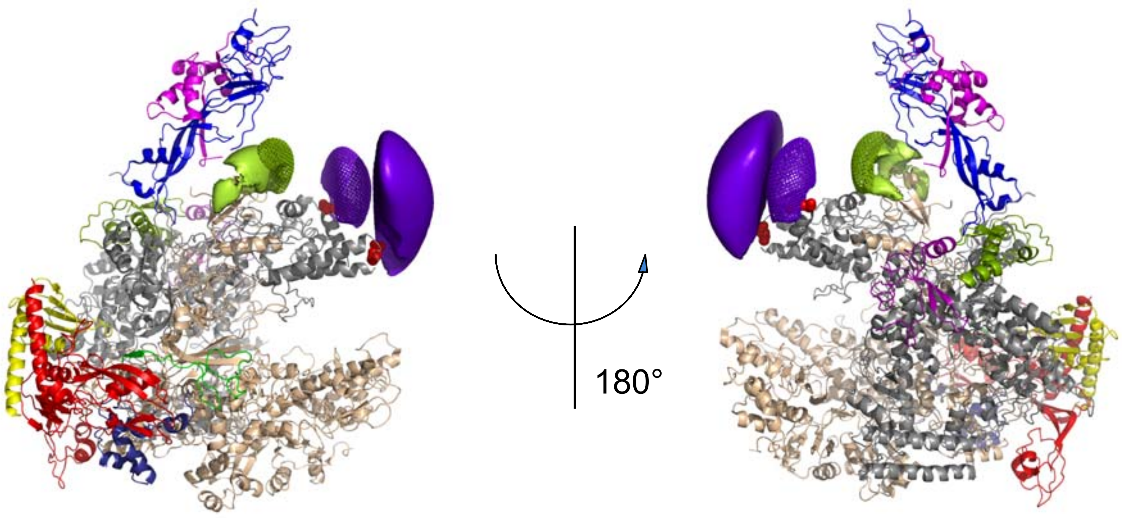

Figure S6. Inferred Primary and Alternative Locations of the TFE WH- and ZR-Domains in Comparison to the RNAP Binding Sites of Eukaryotic TFIIE

The most probable position of the fluorescent probe attached to residue 44 in the TFE WH- domain (large purple surface) was computed from the combination of the main peaks of the histograms (Figure S3). An alternative but less probable position of the WH-domain was calculated considering only the side peaks of the reference fluorescent probes at Rpo7 position 49, Rpo7 position 65, Rpo5 position 11 and the main peak of the reference fluorescent probe at Rpo2'' position 373 (small purple mesh). The most probable position of the fluorescent probe attached to residue 133 in the TFE ZR-domain (large green surface) was computed from the combination of the main peaks of the histograms (Figure S4). An alternative but less probable position of the ZR-domain was calculated considering only the side peak of the reference fluorescent probes at Rpo7 position 65 and the main peaks of the reference fluorescent probes at Rpo7 position 49, Rpo2'' position 373 and Rpo1' position 257 (small green mesh). The sizes of the density surfaces and meshes corresponds to 68% credible volumes. For comparison the eukaryotic TFIIE X-linking sites on the RNAPII are highlighted as red spheres. Hahn and co-workers derivatised the large RNAPII subunits RPB1 and RPB2 with the photoactivatable crosslinker Bpa, formed PICs and identified the polypeptides that were conjugated to specific incorporation sites (Chen et al., 2007). The

eukaryotic TFIIE factor was cross linked to residues RPB1 His-213 and -286 highlighted as red spheres on the structure of the archaeal RNAP (corresponding to residues Rpo1 Lys-186 and Gln-259 in *S. solfataricus* RNAP, pdb 2PMZ).

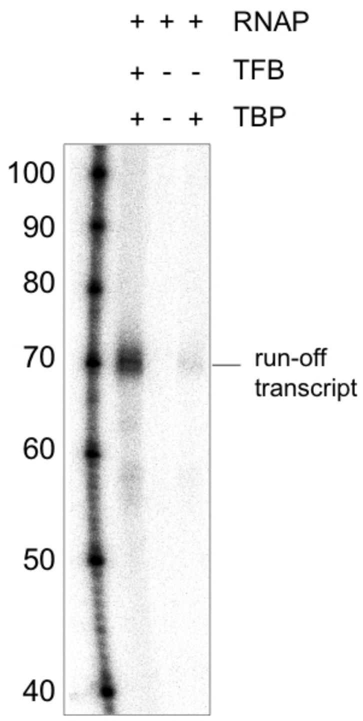

Figure S7. Promoter Directed Transcription by Recombinant *M. jannaschii* RNAP Is Strictly Dependent on Basal Transcription Factors

Promoter directed transcription assays were carried out according to Experimental Procedures. The synthesis of the 70 nt runoff transcript is strictly dependent on the presence of the two transcription factors TBP and TFB.

Table S1. Overview of the Results of the sp-FRET Measurements for the Winged Helix Domain

	Donor (Cy3B)	Acceptor	E_{FRET} [%]	Standard Error [%]	Width	Area [%]	No. of molecules
**A**	TFE	Rpo7-V49_a647	31*	0.34	0.20	83	206
			77	3.36	0.30	17	
**B**	TFE	Rpo7-S65_a647	29*	0.17	0.14	81	197
			56	1.61	0.21	16	
**C**	TFE	Rpo5-K11_a674	29*	0.21	0.15	82	127
			71	1.94	0.22	17	
**D**	TFE	Rpo2''-Q373_dl649	74	0.08	0.11	97	898

*main peak

Table S2. Overview of the Results of the sp-FRET Measurements for the Zinc-Ribbon Domain

	Donor (dl549)	Acceptor	E_{FRET} [%]	Standard Error [%]	Width	Are a	No. of molecules
**A**	TFE	Rpo7-V49_a647	82	0.12	0.12	95	458
**B**	TFE	Rpo7-S65_a647	54	3.03	0.30	21	163
			93*	0.18	0.11	79	
**C**	TFE	Rpo2''-Q373_dl649	33	0.26	0.19	98	369
**D**	TFE	Rpo1'-G257_dl649	69	0.12	0.11	95	499

*main peak

Table S3. Information Used in the NPS Analysis

satellite	attachment point			L_{linker}	d_{linker}	d_{dye}	stdev
	x	y	Z				
Rpo7-V49	143,561	32,085	-39,506	7	4,5	7	1,14
Rpo7-S65	132,473	30,557	-16,165	7	4,5	7	0,84
Rpo5-K11	98,052	79,705	-5,956	7	4,5	7	1,16
Rpo2''-Q373	49,855	-3,639	-7,985	12	4,5	7	1,48
Rpo1'-G257	67,706	21,772	-37,704	12	4,5	7	1,42

All values given in Å.

Table S4. Overview of the Results of the Anisotropy Measurements

Donor	Acceptor	Anisotropy Donor	Anisotropy Acceptor
TFE-G44APA_Cy3B	Rpo7-V49_a647	0,208 ± 0,025	0,243 ± 0,024
	Rpo7-S65_a647		0,198 ± 0,007
	Rpo5-K11_a647		0,288 ± 0,006
	Rpo2’’-Q373_dl649		0,285 ± 0,012
TFE-G133APA_dl549	Rpo7-V49_a647	0,243 ± 0,015	0,243 ± 0,024
	Rpo7-S65_a674		0,192 ± 0,010
	Rpo2’’-Q373_dl649		0,289 ± 0,012
	Rpo1’-G257_dl649		0,262 ± 0,011

Supplemental Experimental Procedures

Production of Labeled TFE and RNAP Derivatives

Protein Production. Amber codons were engineered into genes encoding C-terminally hexahistidine-tagged M. jannaschii TFE (positions 44, 108 and 133) and RNAP subunits Rpo1' (position 257) and Rpo2" (position 373) using SOE (splice by overlap extension) PCR. Genes were expressed in a derivative of Escherichia coli strain BL21(DE3) carrying a plasmid encoding an amber-suppressor tRNA (tRNATyrCUA) and an engineered tyrosyl-tRNA synthethase from *M. jannaschii* (Chin et al., 2002). 10 ml cultures were grown in LB medium containing 100 µg/ml ampicillin and 25 µg/ml tetracycline. 16 h after inoculation cells were pelleted and the pellet was resuspended in 50 ml of M9 minimal medium supplemented with 0.4% glucose, 2mM $MgCl_2$, 0.1 M $CaCl_2$, 3nM $(NH_4)_6Mo_7O_{24}$, 400 nM H_3BO_3, 30 nM $CoCl_2$, 10 nM $CuS0_4$, 80 nM $MnCl_2$, 20 nM $ZnSO_4$, 2mg/ml thiamine, 0.4 mg/ml choline chloride, 0.5 mg/ml folic acid, 0.5 mg/ml nicotinamide, 1 mg/ml myo-inositol, 1 mg/ml pyridoxal, 0.05 mg/ml riboflavin, 1 mg/ml biotin and antibiotics (ampicillin and tetracycline). Cells were grown for another 24h and pelleted (4000 g, 15 min, 4°C). The cell pellet was resuspended in 5 ml of minimal medium and expanded in 500 ml M9 minimal medium (with the supplements as described above). At an optical density of 0.6–0.8 at 600 nm, 1 mM IPTG was added to induce protein expression. The cultures were grown for 6h after induction in the presence of 1 mM of the unnatural amino acid *p*-azido-L-phenylalanine (Chem-Impex international). The cells were harvested by centrifugation and lysed in a buffer containing containing urea (8M), Tris/HCl (50 mM, pH 7.5), and NaCl (250 mM) and subjected to a freeze/thaw cycle and TFE, Rpo1', Rpo2" and TFE were purified by nickel-affinity chromatography on a 1ml Ni-NTA column (GE Healthcare) following the manufacturer's protocols. The target protein was eluted in a buffer containing urea (8M), Tris/HCl (50 mM, pH 7.5), NaCl (250 mM) and 250 mM imidazole. TFE derivatives were renaturated on-column using a linear gradient from 6 M to 0 M urea in a buffer containing 250 mM NaCl, 50 mM Tris-/HCl pH7.5, 100 mM $ZnSO_4$, 30%

glycerol, 20 mM imidazole) and subsequently eluted in the same buffer containing 250 mM imidazole. Typical yields were 4 mg/l expression culture for TFE derivatives and 4-7 mg/l expression for RNAP-subunit derivatives Rpo1'257 and Rpo2"373. Rpo5-11C and Rpo7-49C and -65C derivatives respectively, were prepared as described in (Grohmann et al., 2009).

Protein Chemical Modification. TFE and RNAP-subunit derivatives containing site-specifically incorporated p-azido-L-phenylalanine were labeled by Staudinger ligation using phosphine derivatives of fluorescent probes (Chakraborty et al., 2010; Kiick et al., 2002). TFE derivatives were labeled using Cy3B-phosphine (Chakraborty et al., 2010); RNAP-subunit derivates were labeled with DL649-phosphine (Pierce). Following reaction of 0.5 mg TFE derivative or 6 mg of RNAP-subunit derivative with a 5-fold molar excess of fluorophore-phosphine for 2-16 h at 37°C, beta-mercaptoethanol was added to 1 mM, and, in the case of TFE derivatives, the product was isolated and refolded on a 1-ml Ni-NTA column (GE Healthcare; methods for isolation and refolding as in preceding section). Labelling efficiencies were ~50% for TFE derivatives and ~10-40% for RNAP-subunit derivatives. Rpo5 and Rpo7 derivatives were labeled as described in (Grohmann et al., 2009).

In vitro reconstitution of M.jannaschii RNAP including fluorescently labeled RNAP subunits. Fluorescently labeled RNAP subunits were directly introduced into RNAP reconstitution reactions following protocols described earlier (Werner and Weinzierl, 2002). The excess of non-coupled fluorophore-phosphine was removed during the dialysis steps of the reconstitution process and additionally by size-fractionation the RNAP in a gel filtration run (Superose 6, GE Healthcare).

Nanopositioning System Experiments

Determination of the winged helix and the zinc-ribbon domain of TFE by using NPS

The X-ray structure of the archaeal polymerase of *S. solfataricus* (Hirata et al., 2008) was used as a reference frame for the position calculation. Moreover,

the volume occupied in the crystal structure was used as a restriction for the possible positions of the dye molecules. We assumed zero probability density within an already occupied volume, which was the volume of the protein shrunk by 1 Å to account for uncertainties in the x-ray structure, and equal probability density elsewhere in order to calculate the ADM prior.

The recently developed NPS method (software freely available at http://www.cup.uni-muenchen.de/pc/michaelis/) was applied separately for the two different positions of TFE using the measured FRET efficiencies and the information about the possible SDM positions and Förster distances as described above. As a result we obtain the probability density function for the winged helix and the zinc-ribbon domain of TFE. From this we calculate the smallest volumes that enclose a certain probability, so-called credible volumes. The surface of the credible volume was displayed by using the interactive visualisation program PyMOL.

Determination of the isotropic Förster radius

For each donor-acceptor pair the isotropic Förster radius R_0^{iso} was determined using standard procedures (Vamosi et al., 1996). First, the quantum yield of the donor sample was determined using Rhodamine 101 dissolved in ethanol as a standard (QY = 100 %). Second, overlap integrals were calculated from recorded donor emission spectra (528 to 700 nm with an excitation wavelength of 523 nm) and acceptor absorption spectra (400 to 700 nm). The winged helix domain of TFE was labelled at position G44APA with the donor dye Cy3B. The quantum yield of the donor was determined to 71 % and the calculated isotropic Förster radius R_0^{iso} resulted in 69 Å. The zinc-ribbon domain TFE-G133APA was labelled with the donor dye DyLight549. We assume a fluorescence quantum yield of 6 % (http://www.thermo.com/eThermo/CMA/PDFs/Various/File_9349.pdf) and calculated the isotropic Förster radius R_0^{iso} of 45 Å.

In order to account for uncertainties in the Förster distance due to orientation effects we then measured the steady state fluorescence anisotropies of the donor and acceptor dyes for all attachment sites (results shown in Table 3). Assuming that there is no additional rotational movement beyond the time scale of the fluorescence lifetime, Monte Carlo Simulations were performed in order to calculate the probability densities of the Förster distances assuming

an isotropic distribution of the average dye molecule orientation (Muschielok et al., 2008). For all donor-acceptor pairs the calculated probabilities were fitted using a sum of 10 Gaussian distributions and used in the NPS analysis as described previously (Muschielok et al., 2008).

Experimental setup for sp-FRET, data collection and analysis

All sp-FRET experiments were performed on a homebuilt prism-based total internal reflection fluorescence microscope (TIRFM) described previously (Andrecka et al., 2008). Briefly, a frequency-doubled Nd:YAG laser (532 nm, Spectra-Physics) was used for the excitation of donor molecules and a He-Ne laser (637 nm, Coherent) for the direct excitation of the acceptor molecules. The fluorescence signal of donor and acceptor was combined spatially by the use of a dichroic mirror. Fluorescence intensity was collected through a water-immersion objective (Plan Apo 60X, NA 1,2, Nikon) and directed to an EMCCD camera (iXon DU-897E-CS0-BV, Andor). PIC complexes were immobilised onto the surface of a microfluidic chamber surface via PEG-Biotin/Neutravidin/Biotin as described previously (Andrecka et al., 2008). All measurements were recorded with an exposure time of 100 ms per frame for the duration time of 40 s. The acquired data was analysed using custom-written MATLAB software. We used a fully automated routine to find FRET pairs using an intensity threshold for the acceptor signal during the FRET measurement. The algorithm then calculates and subtracts the local background and computes fluorescence trajectories (Andrecka et al., 2008). For the calculation of the FRET efficiency of each individual FRET pair, we used the following formula: $E = \frac{I_A - \beta \cdot I_D}{I_A + \gamma \cdot I_D}$, where $\gamma = \frac{\Delta I_A}{\Delta I_D} = \frac{I_A - I_A^{'}}{I_D^{'} - I_D}$ and $\beta = \frac{I_A^{'}}{I_D^{'}}$

I_A and I_D are the background corrected intensities from donor and acceptor channels, and I and I´ are intensities before and after acceptor photobleaching process, respectively. The parameters β and γ are experimental correction factors, where β accounts for the leakage of the donor emission into the acceptor channel, and γ includes the fluorescence quantum yields of the fluorophores and the detection efficiencies of the two channels. We determined the correction factors for all FRET pairs individually by time averaging the intensities I and I´. In order to determine the γ-factor individually for each FRET pair only data where acceptor photobleaching occurred prior to

donor photobleaching were used in the data analysis (Andrecka et al., 2008). As a result no zero FRET peak is visible in the histograms. The resulting histograms were computed either for every time point (frame-wise histogram) or using the determined mean FRET efficiencies of every molecule (molecule-wise histogram). The histograms were then fitted with one or more Gaussian distributions and the mean FRET efficiency and its standard error were determined from the fit (see Table 1 and 2). These results were then used for further analysis with NPS (Muschielok et al., 2008).

Uncertainty in the position of dye molecules attached to known positions

Satellite dye molecules (SDMs) were attached to known positions within the archaeal polymerase using flexible linkers. While the attachment point is known from the x-ray structure of the archaeal polymerase of *S. solfataricus* (pdb file: 2pmz) (Hirata et al., 2008), the precise location of the dye molecule is not. For the NPS analysis we therefore calculated the volume that is sterically accessible to the dye molecules, given the point of attachment, size of the dye molecule and the linker length (Muschielok et al., 2008). To this end, the SDMs were approximated by a sphere of diameter d_{dye} and linked to the protein complexes by flexible linkers of dimensions L_{linker} and d_{linker} (see Table **S3**). We assume each SDM position within this accessible volume equally probable and for calculation purposes approximate the resulting probability density function by a sum of 10 Gaussian distributions. These Gaussians are used in the NPS analysis to describe the uncertainty of the SDM position (Muschielok et al., 2008) (see Figure **S2**).

Supplemental References

Andrecka, J., Lewis, R., Bruckner, F., Lehmann, E., Cramer, P., and Michaelis, J. (2008). Single-molecule tracking of mRNA exiting from RNA polymerase II. Proc Natl Acad Sci U S A *105*, 135-140.

Chakraborty, A., Wang, D., Ebright, Y.W., and Ebright, R.H. (2010). Azide-specific labeling of biomolecules by Staudinger-Bertozzi ligation phosphine derivatives of fluorescent probes suitable for single-molecule fluorescence spectroscopy. Methods Enzymol *472*, 19-30.

Chen, H.T., Warfield, L., and Hahn, S. (2007). The positions of TFIIF and TFIIE in the RNA polymerase II transcription preinitiation complex. Nat Struct Mol Biol *14*, 696-703.

Chin, J.W., Santoro, S.W., Martin, A.B., King, D.S., Wang, L., and Schultz, P.G. (2002). Addition of p-azido-L-phenylalanine to the genetic code of Escherichia coli. J Am Chem Soc *124*, 9026-9027.

Grohmann, D., Hirtreiter, A., and Werner, F. (2009). RNAP subunits F/E (RPB4/7) are stably associated with archaeal RNA polymerase: using fluorescence anisotropy to monitor RNAP assembly in vitro. Biochem J *421*, 339-343.

Hirata, A., Klein, B.J., and Murakami, K.S. (2008). The X-ray crystal structure of RNA polymerase from Archaea. Nature *451*, 851-854.

Humphrey, W., Dalke, A., and Schulten, K. (1996). VMD: visual molecular dynamics. J Mol Graph *14*, 33-38, 27-38.

Kiick, K.L., Saxon, E., Tirrell, D.A., and Bertozzi, C.R. (2002). Incorporation of azides into recombinant proteins for chemoselective modification by the Staudinger ligation. Proc Natl Acad Sci U S A *99*, 19-24.

Littlefield, O., Korkhin, Y., and Sigler, P.B. (1999). The structural basis for the oriented assembly of a TBP/TFB/promoter complex. Proc Natl Acad Sci U S A *96*, 13668-13673.

Muschielok, A., Andrecka, J., Jawhari, A., Bruckner, F., Cramer, P., and Michaelis, J. (2008). A nano-positioning system for macromolecular structural analysis. Nat Methods *5*, 965-971.

Vamosi, G., Gohlke, C., and Clegg, R.M. (1996). Fluorescence characteristics of 5-carboxytetramethylrhodamine linked covalently to the 5' end of oligonucleotides: multiple conformers of single-stranded and double-stranded dye-DNA complexes. Biophys J *71*, 972-994.

Werner, F., and Weinzierl, R.O. (2002). A recombinant RNA polymerase II-like enzyme capable of promoter-specific transcription. Mol Cell *10*, 635-646.
